# Clinical Parameters and Oral Fluid Biomarkers in Gingivitis Subjects using an Electric Toothbrush with Irrigator vs a Manual Toothbrush Alone over 8 Weeks: A Randomised Controlled Clinical Trial

**DOI:** 10.3290/j.ohpd.b966767

**Published:** 2021-02-19

**Authors:** Christoph A. Ramseier, Chloé Petitat, Sidonia Trepp, Niklaus P. Lang, Sigrun Eick, Ralf Adam, Renzo A. Ccahuana-Vasquez, Matthew L. Barker, Hans Timm, Malgorzata Klukowska, Giovanni E. Salvi

**Affiliations:** a Dentist, Department of Periodontology, School of Dental Medicine, University of Bern, Switzerland. Contributed to study concept and design, material preparation, data collection, wrote first draft of the manuscript, edited, read, and approved the final manuscript.; b Dentist, Department of Periodontology, School of Dental Medicine, University of Bern, Switzerland. Material preparation, data collection, wrote first draft of the manuscript, read and approved the final manuscript.; c Professor Emeritus, Department of Periodontology, School of Dental Medicine, University of Bern, Switzerland. Edited, read, and approved the final manuscript.; d Associate Professor, Department of Periodontology, School of Dental Medicine, University of Bern, Switzerland. Contributed to study concept and design, read and approved the final manuscript.; e Research Fellow (RA); Senior Clinical Scientist-Dentist (RAC-V), Procter & Gamble Service GmbH, Kronberg, Germany. Contributed to study concept and design, read and approved the final manuscript.; f Statistician, Procter & Gamble Company, Mason, OH, USA. Statistical analysis, edited, read and approved the final manuscript.; g Clinical Trial Manager, Procter & Gamble Service GmbH, Kronberg, Germany. Contributed to study concept and design, material preparation and data collection, edited the manuscript, read, and approved the final manuscript.; h Clinical Scientist-Dentist, Procter & Gamble Company, Mason, OH, USA. Contributed to study concept and design, read and approved the final manuscript.; i Associate Professor, Department of Periodontology, School of Dental Medicine, University of Bern, Switzerland. Read and approved the final manuscript.

**Keywords:** gingival crevicular fluid, gingivitis, prevention, toothbrushing

## Abstract

**Purpose:**

To compare clinical outcomes and oral fluid biomarkers in gingivitis subjects using an electric toothbrush/irrigator combination (test) or a manual toothbrush alone (control) over 8 weeks.

**Materials and Methods:**

Subjects were randomly assigned to two groups of n = 30. In both groups, toothbrushing was performed twice daily at home and no additional interdental cleaning aids were allowed. Plaque Index (PLI), Gingival Index (GI), whole saliva (WS), and gingival crevicular fluid (GCF) samples were collected at weeks 2, 4, and 8.

**Results:**

Subjects’ mean age was 23 years and 52% were female. Overall baseline means were 1.31 for PLI, 1.07 for GI, and 34.9 for number of bleeding sites. At every follow-up visit, both groups differed statistically significantly (p < 0.001) from baseline for all clinical parameters. The test group demonstrated statistically significantly (p < 0.001) greater reductions in GI vs the control group by 18% at week 2, 17% at week 4 and 24% at week 8. The test group also demonstrated statistically significantly (p < 0.002) greater reductions in the number of bleeding sites vs the control group by 33% at week 2, 34% at week 4 and 43% at week 8. Between-group comparisons for both WS and GCF revealed numerical trends for decreased levels of interleukin (IL)-1β in GCF after 4 and 8 weeks, but these were not statistically significant.

**Conclusion:**

In subjects using the electric toothbrush/irrigator combination, increased clinical improvements may be found accompanied by similarly improved trends for oral fluid biomarkers such as IL-1β.

The most prevalent oral diseases include dental caries and periodontal disease. While the former is a destruction of the oral hard tissue due to production of acids from metabolising low molecular-weight carbohydrates, the latter is a group of inflammatory conditions affecting the supporting structures of the dentition.^1^ The impact of dental biofilms on both caries and periodontal diseases has been well studied.

Periodontal diseases are further divided into reversible and non-reversible categories. Gingivitis is the reversible inflammatory response of the marginal gingiva to dental biofilm. Periodontitis, the destructive category of periodontal disease, is a non-reversible inflammatory state of the supporting structures. After its initiation, the disease progresses with the loss of attachment to the root surface, resorption of alveolar bone, and the formation of periodontal pockets. If left untreated, the disease continues with progressive alveolar bone destruction, leading to increased tooth mobility and subsequent tooth loss.^16^

Periodontitis is the most prevalent form of destructive periodontal disease, causing a public health burden in all populations worldwide. Data from 2009 to 2010 indicates that approximately half of US adults (age 30 years and older) had periodontitis.^5^ Further breakdown of these findings indicate that approximately 10% are affected by severe periodontitis.^7^ In earlier studies by Schätzle et al^21^ and Lang et al,^11^ gingivitis was established as a risk factor for chronic periodontitis. While gingivitis always precedes periodontitis, not all gingival sites presenting gingivitis proceed to periodontitis.^15^ Consequently, successful prevention of gingivitis can facilitate the prevention of periodontitis.^3^ Indeed, in support of professional care at the dental practice, daily self-administered oral hygiene efforts can successfully prevent gingivitis.^18-20,24-27^

A study by Ramseier et al^17^ investigated the potential of whole saliva (WS) and periodontal pathogens for the diagnosis of periodontal diseases in a cohort of 100 subjects. Based on clinical and radiographic data, these subjects were divided into four different subgroups: periodontal health (BOP ≤20%), gingivitis (BOP >20%), mild chronic periodontitis (≤30% of dental sites with clinical attachment loss >3 mm), and moderate to severe chronic periodontitis (>30% dental sites with clinical attachment loss >3 mm). Interestingly, elevated levels of both matrix metalloproteinase (MMP)-8 and *Treponema denticola* were determined between a smaller group of 18 subjects with gingival health and 32 subjects with gingivitis.

The potential to identify differences in oral fluid biomarkers between individuals using different oral hygiene regimens might be both scientifically and clinically relevant. Therefore, the primary objective of this study was to investigate whether the use of an enhanced oral hygiene regimen that included a combination of electric toothbrush and an irrigator yielded improved gingival health in terms of clinical measurement of plaque and gingivitis, when compared to a standard homecare oral hygiene regimen (i.e. regular manual toothbrushing alone) over a period of 8 weeks. A secondary objective was to determine if oral fluid biomarker signatures correlated with clinical outcomes.

## Materials and Methods

### Study Population

The study protocol was submitted to and approved by the Ethics Committee of the Canton of Bern, Switzerland (number KEK-BE: 066/11). The study was registered in the ISRCTN registry (http://www.isrctn.com) under the ID: ISRCTN15360464.

A total of 60 healthy adult volunteers presenting with gingivitis and without the presence of probing depths >4 mm were enrolled and randomly assigned to a test (n = 30) and a control group (n = 30), respectively.

### Sample Size Calculation

The number of subjects was determined following a power calculation based on reduction in bleeding on probing with data available from previously published research.^9,10^ With a sample of 27 subjects per group, the study was estimated to have 90% power to demonstrate that 8 weeks following the baseline visit, a statistically significant difference would be found using a two-sided 5% significance level. To compensate for an estimated drop-out rate of 10%, 30 subjects per group were enrolled (60 subjects in total).

### Inclusion and Exclusion Criteria

An advertisement in the local newspaper was used for the recruitment of volunteers. To be included in the study, the subjects had to be at least 18 years of age, provide written informed consent, have at least 20 gradable natural teeth presenting with 15 or more gingival bleeding sites, and be in good general health based on a review of their medical history. Included subjects additionally agreed to 1) return for the clinical visits 2, 4, and 8 weeks following the baseline examination, 2) avoid any non-study based oral hygiene measures (including dental floss), and 3) delay any elective professional oral care such as dental prophylaxis outside of the study protocol.

Following screening, subjects were excluded if there was 1) evidence of periodontitis, 2) advanced gingival recession of > 3 mm, 3) active treatment for periodontitis, 4) fixed facial or lingual orthodontic appliances, 5) prior regular use of an electric toothbrush, 6) need for antibiotic prophylaxis prior to dental visits, 7) use of antibiotic or prescription mouthrinses within one month prior to screening, 8) dental prophylaxis within one month prior to screening, 9) any diseases or conditions that could be expected to interfere with the subject safely completing the study, and 10) pregnancy or lactation.

During the study, subjects were excluded if they 1) used antibiotics, 2) used any non-study oral hygiene devices (including dental floss), 3) participated in any other clinical study, and 4) received dental prophylaxis (outside of the protocol) or other elective dental therapy since the previous visit.

### Randomisation Method (Balance and Assignment System)

Following the baseline examination, subjects were stratified based on age, tobacco use, number of bleeding sites, and mean PLI. Within these strata, subjects were randomly assigned to one of the groups using software. Subjects residing in the same household were assigned to the same group.

To maintain blinding randomisation and product distribution were performed in areas separate from the clinical examiner (CAR).

### At-home Oral Hygiene Protocols

For the eight-week duration of the study, the subjects used the study products at home in place of their normal oral hygiene products. According to the verbal and written instructions provided with their devices, the subjects used these devices twice daily. In both groups, the subjects were asked to refrain from flossing for the entire eight weeks.

The test group used the electric toothbrush/irrigator center (Oral-B Professional Care Oxyjet 1000 Center with Oral-B Precision Clean brush head EB20) and regular toothpaste (Blend-a-Med Classic, sodium fluoride, 1450 ppm F^-^). The subjects brushed their teeth using the electric toothbrush with toothpaste according to manufacturer’s instructions. Then subjects were instructed to rinse their mouth with the water irrigator according to manufacturer’s instructions with 300 ml water for approximately 1 min.

The control group used the manual toothbrush (Oral B Indicator 35 soft) and regular toothpaste (Blend-a-Med Classic, sodium fluoride, 1450 ppm F^-^). The subjects brushed their teeth twice daily in their customary manner while using the products provided.

### Clinical Parameters

The subjects attended clinical visits at baseline, and again at 2, 4, and 8 weeks. Continuance criteria were verified at every clinical visit, including confirmation that subjects refrained from brushing their teeth and performing any other oral hygiene procedures 12 h prior to the visit and refrained from eating, chewing gum, drinking, and using tobacco for 4 h prior to the visit.

1. Unstimulated WS was collected by passive drooling into sterile plastic tubes from all the subjects. The collection was completed as soon as 2 ml of WS had been collected or a maximum of 15 min of sampling time was reached. Subsequently, the samples were placed on ice, aliquoted and supplemented with a proteinase inhibitor cocktail prior to storage at -79°C.
2. Plaque index (PLI): The plaque deposits on the teeth were scored.^22^ The PLI evaluates plaque in contact with the gingival margin on six surfaces of all teeth (i.e. distobuccal, buccal, mesiobuccal, distolingual, lingual and mesiolingual).
3. Subsequently, GCF samples were collected using standardised filter paper strips (Periopaper, Harco; Winnipeg, Manitoba, Canada). The paper strips were placed at the entrance of the sulcus for 30 s at the selected test sites. Samples were obtained from 2 sites in the first quadrant (mesiobuccally on 16 and 17). If target teeth were missing, the 2 most posterior teeth in the first quadrant were chosen. Selected sites were first isolated with cotton rolls and air dried. Following collection, the paper strip was placed in a dry Eppendorf tube (1.5 ml natural flat cap DNase- and RNase-free microcentrifuge tubes, Starlab; Ahrensburg, Germany) and then stored at -79°C until processed.
4. Gingival index (GI): GI was used to evaluate gingival health or disease.^13^ The entire dentition, with the exception of the third molars, was evaluated. For each tooth, six gingival areas (distobuccal, buccal, mesiobuccal, mesiolingual, lingual, and distolingual) were scored using adequate light, a mouth mirror, a periodontal probe, and compressed air.

Prior to leaving the clinic, the subjects were scheduled for their next visit and reminded to refrain from brushing their teeth for 12 h and to refrain from eating, chewing gum, drinking, and using tobacco for 4 h prior to their next visit. Subjects also received a text message via cellphone to remind them to comply with these requirements.

### Analysis of WS and Gingival Crevicular Fluid (GCF)

WS and GCF were quantitatively analysed for the amount of matrix-metalloproteinase (MMP)-3, MMP-8, and interleukin (IL)-1β by means of commercially available enzyme-linked immunosorbent assay (ELISA) kits (R&D Systems Europe; Abingdon, UK) according to the manufacturer’s instructions.

### Statistical Analysis

The statistical analysis was performed using SAS Institute v9.3 software for Windows (Cary, NC, USA; 2014). Summary statistics (e.g. means, standard deviations, frequencies, etc.) of the demographic characteristics and each measurement were calculated for both groups and visits. Comparisons between groups for 1) examiner-graded indices and 2) oral fluid biomarker concentration levels at baseline, 2, 4, and 8 weeks were performed. For examiner-graded indices, the two groups were compared using the ANCOVA method with baseline as a covariate. The null hypothesis of no change of the primary outcome variable GI was tested and p-values were adjusted for multiple comparisons using Holm-Bonferroni tests. For oral fluid biomarker concentration levels, data were analysed on the natural log scale using ANCOVA to compare groups with baseline. Values less than the lower quantification limit (cut-off) were assigned a value of half the lower limit (e.g. values below a lower limit of 25 were assigned a value of 12.5). For each measurement and group, comparisons to baseline for the post-baseline visits were made utilising paired-difference t-tests. Statistical tests were two-sided using a significance level at p < 0.05.

## Results

### Study Sample

A total of 82 subjects were enrolled in the study. There were 21 screening failures and one subject dropped out prior to randomisation. Subsequently, 60 subjects were randomly assigned to either the test or control group. All of the 60 subjects completed the study.

The subjects’ age ranged between 19 and 36 years, with a mean age of 22.8 years. There were 31 (52%) females and 29 (48%) males (Table 1). All subjects were Caucasian. There were 3 subjects who missed their week-2 visit. Three subjects took antibiotics ahead of their week-4 visit and were therefore excluded from analysis.

**Table 1:** Baseline demographic summary

	Test (n = 30)	Control (n = 30)	Overall (n = 60)	p-value
**Age (years)**				
Mean (SD)	23.1 (3.65)	22.4 (2.40)	22.8 (3.08)	0.4069^a^
Min–Max	19–36	19–29	19–36	
**Gender**				
Female^b^	17 (57%)	14 (47%)	31 (52%)	0.4383^c^
Male^b^	13 (43%)	16 (53%)	29 (48%)	

^a^Two-sided ANOVA p-value for group comparison. ^b^ Number (percent) of subjects in each category. ^c^ Two-sided chi-squared p-value for group comparison.

### Plaque Index (PLI)

The baseline plaque index (PLI) scores were not statistically significantly different (p = 0.35) between groups, with means of 1.29 and 1.34 for the test and control groups, respectively (Table 2). At week 2, PLI scores were reduced by -0.60 for the test group and -0.45 for the control group. Both reductions were statistically significantly different vs baseline (p < 0.0001). At week 4, PLI reductions were -0.66 for the test group and -0.51 for the control group. Again, both were statistically significantly lower vs baseline (p < 0.0001). At week 8, the PLI reductions were -0.94 for the test group and -0.72 for the control group, both of which were statistically significant different from baseline (p < 0.0001) (Table 2).

**Table 2:** Descriptive summary of the plaque index (PLI) per group and clinical visit

	n	Mean (SD)	Median	Min–Max.	Mean change from baseline (SD)	p-value
**Baseline**						
Control	30	1.34 (0.21)	1.37	0.84–1.61	NA	NA
Test	30	1.29 (0.21)	1.36	0.91–1.60	NA	NA
Overall	60	1.31 (0.21)	1.37	0.84–1.61	NA	NA
**Week 2**						
Control	29	0.90 (0.28)	0.89	0.35–1.39	-0.45 (0.24)	<0.0001
Test	28	0.69 (0.26)	0.65	0.32–1.33	-0.60 (0.27)	<0.0001
**Week 4**						
Control	28	0.82 (0.27)	0.77	0.37–1.41	-0.51 (0.23)	<0.0001
Test	29	0.62 (0.28)	0.53	0.20–1.24	-0.66 (0.28)	<0.0001
**Week 8**						
Control	30	0.62 (0.32)	0.63	0.20–1.63	-0.72 (0.30)	<0.0001
Test	30	0.35 (0.21)	0.34	0.06–0.81	-0.94 (0.23)	<0.0001

SD: standard deviation.

**Fig 1:**
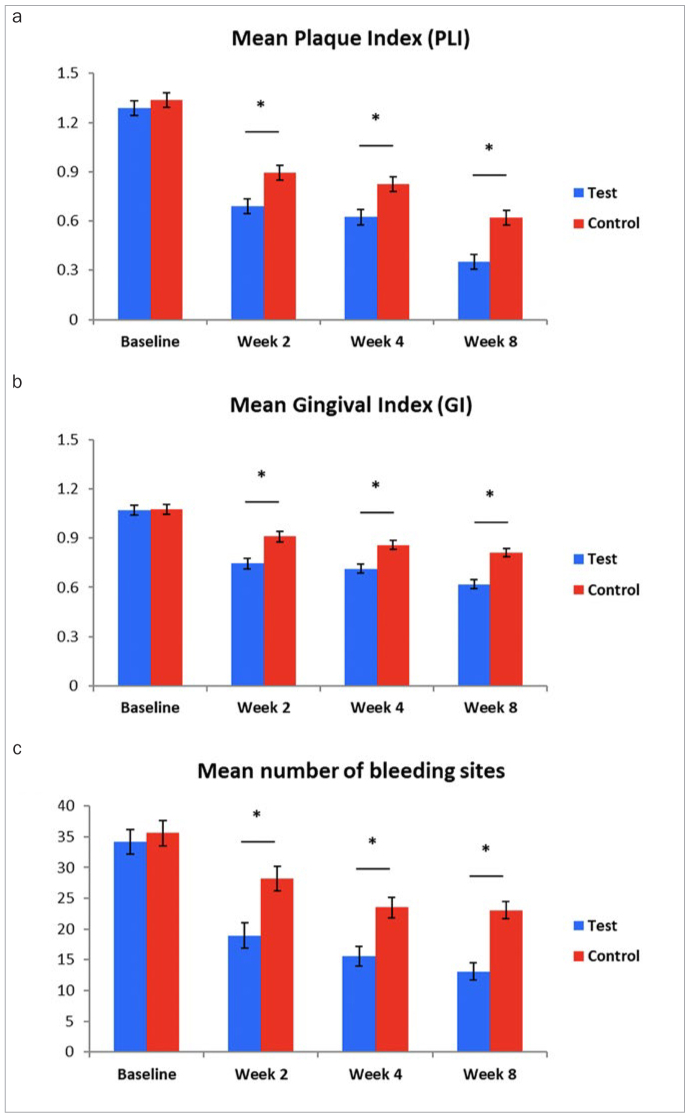
a. Mean plaque index (PLI); b. gingival index (GI); c. number of bleeding sites. n = 30 test subjects and n = 30 control subjects at baseline, 2, 4, and 8 weeks. *statistically significant difference between groups (p < 0.05).

The between-group differences in PLI were all statistically significant, favouring the test group over the control group with adjusted means (SE) as follows: 0.705 (0.046) vs 0.877 (0.045), a 19.7% reduction, with p = 0.01 for week 2; 0.637 (0.046) vs 0.810 (0.047), a 21.4% reduction with p = 0.01 for week 4; and 0.364 (0.046) vs 0.608 (0.046), a 40.0% reduction, with p = 0.0004 for week 8 (Fig 1a). For interproximal sites, the PLI % reduction in adjusted means for the test group relative to control was 16.5% for week 2, 19.3% for week 4, and 37.0% for week 8, with each timepoint demonstrating statistically significant (p≤0.0136) differences between groups.

### Gingival Index (GI)

The baseline GI score was not statistically significantly different (p = 0.88) between groups, with means of 1.07 for each of the test and control groups (Table 3).

**Table 3:** Descriptive summary of the gingival index (GI) per group and clinical visit

	n	Mean (SD)	Median	Min–Max	Mean change from baseline (SD)	p-value
**Baseline**						
Control	30	1.07 (0.14)	1.07	0.85–1.35	NA	NA
Test	30	1.07 (0.10)	1.10	0.83–1.33	NA	NA
Overall	60	1.07 (0.12)	1.10	0.83–1.35	NA	NA
**Week 2**						
Control	29	0.91 (0.22)	0.92	0.46–1.34	-0.16 (0.15)	<0.0001
Test	28	0.74 (0.21)	0.72	0.34–1.35	-0.33 (0.18)	<0.0001
**Week 4**						
Control	28	0.86 (0.16)	0.85	0.59–1.15	-0.21 (0.15)	<0.0001
Test	29	0.72 (0.18)	0.70	0.37–1.07	-0.35 (0.14)	<0.0001
**Week 8**						
Control	30	0.813 (0.18)	0.75	0.58–1.23	-0.26 (0.13)	<0.0001
Test	30	0.618 (0.16)	0.57	0.29–0.95	-0.45 (0.15)	<0.0001

SD: standard deviation.

At week 2, GI reductions were -0.33 for the test group and -0.16 for the control group. At week 4, GI reductions were -0.35 for the test group and -0.21 for the control group. At week 8, GI reductions were -0.45 for the test group and -0.26 for the control group. Both groups demonstrated statistically significant reductions in GI from baseline (p < 0.0001) at each timepoint (see detailed summary statistics in Table 3).

Consistent with changes in PLI, the differences in GI between groups were statistically significant, favouring a reduction in the test group vs control by 18.1% for week 2 (p = 0.0004), 16.7% for week 4 (p = 0.0003), and 23.6% for week 8 (p < 0.0001) with adjusted means (SE) of 0.745 (0.031) vs 0.909 (0.031) for week 2, 0.714 (0.026) vs 0.858 (0.027) for week 4, and 0.619 (0.026) vs 0.811 (0.026) for week 8 (Fig 1b).

For interproximal sites, the % reduction in GI adjusted means for the test group relative to control was 14.6% for week 2, 12.5% for week 4, and 20.2% for week 8. Again, all differences were statistically significant and in favour of the test group (p≤0.0028).

### Number of Bleeding Sites

The baseline number of bleeding sites was not statistically significantly different (p = 0.61) between groups, with a mean of 34.867 (10.362) for the total population.

At week 2, the test group showed a reduction in the number of bleeding sites of 15.8 compared with 6.7 for the control group. At week 4, the test group showed a reduction in the number of bleeding sites of 18.9 compared with 11.6 for the control group. Week 8 reductions in number of bleeding sites were 21.4 for the test group and 12.2 for the control group. All reductions for both groups were statistically significant compared to baseline (p < 0.001) (Fig 1c).

The treatment differences between the groups were 33% for week 2, 34% for week 4, and 43% for week 8, all in favour of the test group and statistically significant (p ≤ 0.0021).

### WS and GCF Samples

**Fig 2:**
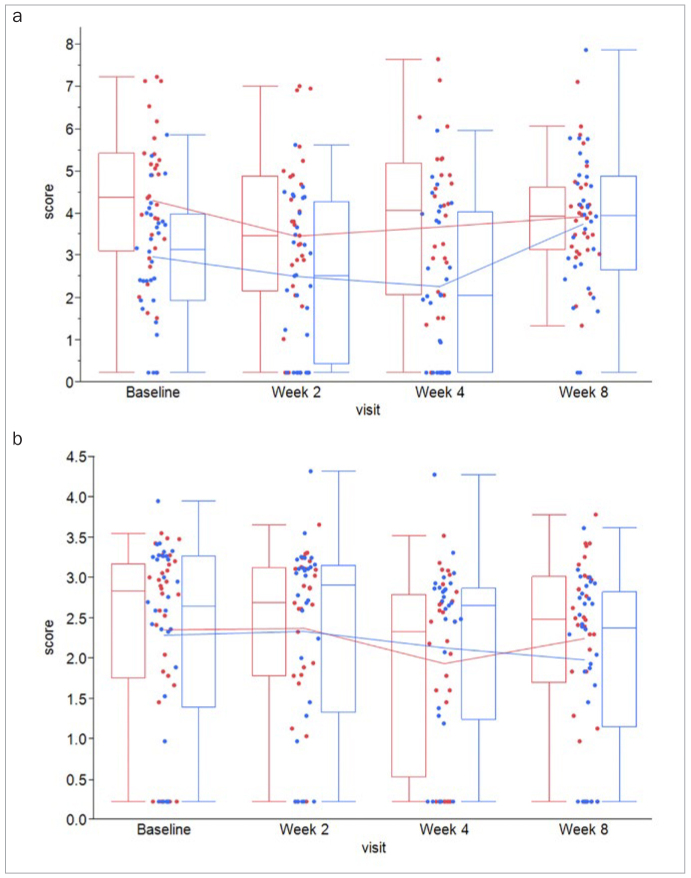
Box-and-whisker plots of (a) MMP-3-levels in whole saliva and (b) gingival crevicular fluid (GCF) from n = 30 test subjects (red) and n = 30 control subjects (blue) collected at the clinical visits at baseline, 2, 4, and 8 weeks.

**Fig 3:**
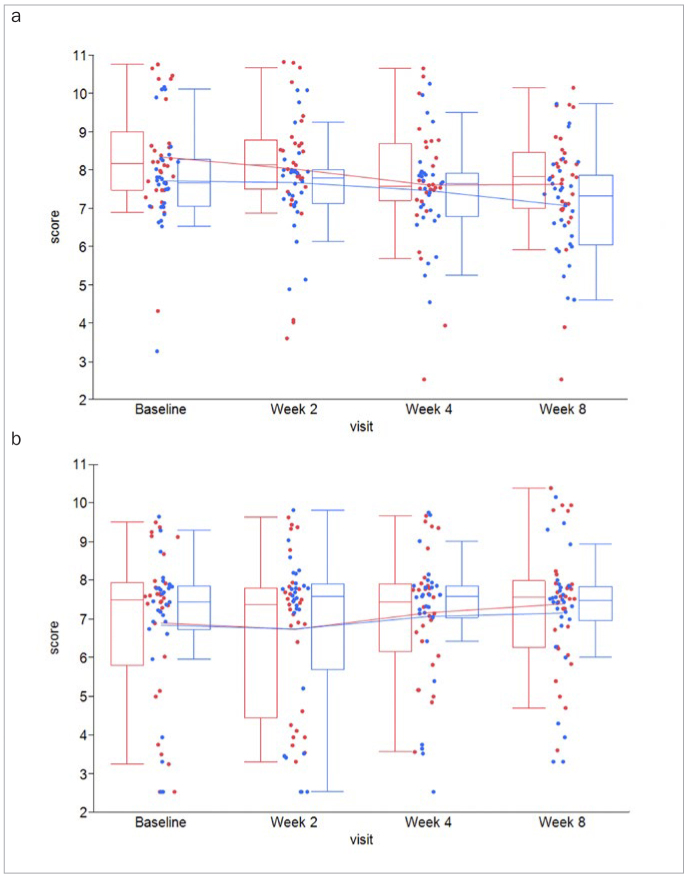
Box-and-whisker plots of (a) MMP-8-levels in whole saliva and (b) gingival crevicular fluid (GCF) from n = 30 test subjects (red) and n = 30 control subjects (blue) collected at the clinical visits at baseline, 2, 4, and 8 weeks.

**Fig 4:**
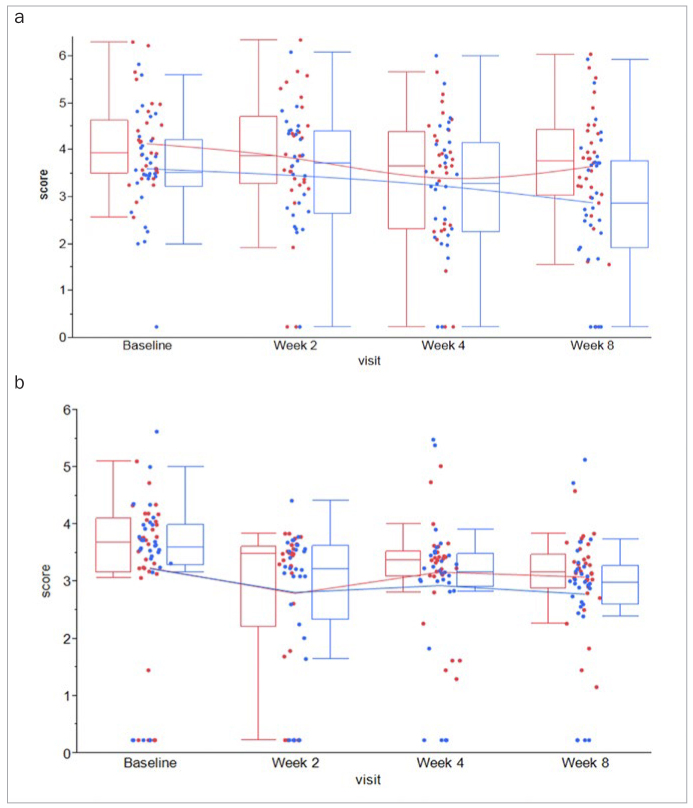
Box-and-whisker plot of (a) IL-1β-levels in whole saliva (WS) and (b) gingival crevicular fluid (GCF) from n = 30 test subjects (blue) and n = 30 control subjects (red) collected at baseline, 2, 4, and 8 weeks.

In both WS and GCF, overall levels of MMP-8 were higher compared to levels of MMP-3 and IL-1β, respectively (Table 4; Figs 2 and 3). Moreover, levels in both groups and all biomarkers remained unaltered over the course of 8 weeks. However, even though numerical trends were found toward lower levels of IL1-β in GCF at weeks 4 and 8 (Fig 4), following Holm-Bonferroni tests for multiple testing, no between-group differences remained statistically significant for any biomarker (Table 4).

**Table 4:** Biomarker levels from whole saliva (WS) and gingival crevicular fluid (GCF) from (n = 30) test and (n = 30) control subjects at weeks 2, 4, and 8

Index	Visit	Group	Adjusted mean (SE) log scale	Adjusted mean (SE) original scale	Between-group difference p-value
**WS**					
IL-1β	Week 2	Control	3.579 (0.236)	35.828 (1.267)	0.9983
		Test	3.579 (0.238)	35.855 (1.268)	
	Week 4	Control	3.224 (0.237)	25.125 (1.267)	0.6530
		Test	3.373 (0.229)	29.158 (1.257)	
	Week 8	Control	3.513 (0.234)	33.564 (1.264)	0.1719
		Test	3.058 (0.231)	21.287 (1.260)	
MMP-3	Week 2	Control	2.952 (0.241)	19.150 (1.272)	0.6717
		Test	3.101 (0.252)	22.213 (1.286)	
	Week 4	Control	3.270 (0.322)	26.302 (1.380)	0.2317
		Test	2.718 (0.323)	15.152 (1.381)	
	Week 8	Control	3.955 (0.275)	52.184 (1.317)	0.7288
		Test	4.092 (0.283)	59.876 (1.327)	
MMP-8	Week 2	Control	7.716 (0.189)	2243.5 (1.209)	0.5653
		Test	7.872 (0.193)	2624.0 (1.213)	
	Week 4	Control	7.362 (0.197)	1574.4 (1.217)	0.3098
		Test	7.644 (0.193)	2089.0 (1.213)	
	Week 8	Control	7.367 (0.186)	1582.7 (1.205)	0.7708
		Test	7.290 (0.187)	1465.2 (1.205)	
**GCF**					
IL-1β	Week 2	Control	2.793 (0.133)	16.335 (1.142)	0.8698
		Test	2.824 (0.135)	16.852 (1.145)	
	Week 4	Control	3.167 (0.082)	23.735 (1.086)	0.0605
		Test	2.946 (0.081)	19.026 (1.084)	
	Week 8	Control	3.083 (0.093)	21.830 (1.098)	0.0313 (n.s.)
		Test	2.792 (0.093)	16.317 (1.098)	
MMP-3	Week 2	Control	2.343 (0.105)	10.408 (1.111)	0.8103
		Test	2.379 (0.107)	10.791 (1.113)	
	Week 4	Control	1.923 (0.144)	6.839 (1.155)	0.2671
		Test	2.149 (0.141)	8.576 (1.152)	
	Week 8	Control	2.230 (0.124)	9.295 (1.132)	0.2171
		Test	2.010 (0.124)	7.463 (1.132)	
MMP-8	Week 2	Control	6.727 (0.241)	834.68 (1.273)	0.8904
		Test	6.775 (0.245)	875.37 (1.278)	
	Week 4	Control	7.179 (0.113)	1311.9 (1.120)	0.5069
		Test	7.073 (0.111)	1179.8 (1.118)	
	Week 8	Control	7.383 (0.143)	1608.8 (1.154)	0.3310
		Test	7.185 (0.143)	1319.4 (1.154)	

SE: standard error; ns: not statistically significant following the Holm-Bonferroni correction for multiple testing (p = 0.0313 > alpha = 0.05/4 = 0.0125).

## Discussion

The outcomes demonstrated that the use of an oscillating-rotating electric toothbrush with an irrigator resulted in lower levels of plaque and improved gingival health after an 8-week period of use compared with the use of a regular manual toothbrush alone. Between-group differences of all clinical parameters were statistically significant at weeks 2, 4, and 8. Similar numerical trends were demonstrated with decreased levels of oral fluid biomarkers at week 8, although between-group comparisons did not differ statistically significantly.

While the majority of the population still uses traditional manual toothbrushes, electric toothbrushes have become more widely used today. Whereas a few decades ago, the efficacy and effectiveness of electric toothbrushes was still being evaluated, research has meanwhile established that in comparison to manual toothbrushes, oscillating-rotating power toothbrushes lead to superior full-mouth and interproximal plaque removal and reduction of gingivitis. Additionally, the use of an irrigator has been shown to improve oral health.^6,18,19,25-27^ In keeping with this evidence, the results of the present study indicate that the electric toothbrush with an irrigator performed better than the manual toothbrush in the interproximal areas. Consequently, the host’s immune response may be reflected in the oral fluid biomarker analysis, as initially hypothesised.

No statistically significant between-group differences were found for oral fluid biomarkers collected at each clinical visit. Overall, data collection in the present study was well standardised. All clinical examinations were performed by one examiner (CAR). Moreover, since both treatment groups in this study used a toothbrush (electric or manual) twice per day for the period of 8 weeks, levels of oral fluid biomarkers indicative of disease were expected to be generally low.

Although clinical data from this study may reflect ideal conditions for the interpretation of oral fluid biomarkers, these results slightly differed from the findings presented by other studies in this field. A number of studies assessing the development of gingivitis and the restoration of gingival health have used the classical experimental gingivitis model as first described by Löe et al,^14^ in which the absence of oral hygiene resulted in a significant host immune response when compared to regular oral hygiene. Using their model, previous studies aimed to detect the specific biomarker and microbial signatures to distinguish between gingivitis and gingival health. In a recent experimental gingivitis study by Lee et al,^12^ higher levels of IL-6 and MMP-1 at baseline demonstrated the strongest ability to predict the development of gingivitis. In the present study, however, MMP-8, MMP-3, and IL-1β were chosen as the key inflammatory biomarkers aiming to detect gingival improvements. Out of these, only IL-1β from GCF was able to demonstrate a statistically significant difference after 8 weeks of observation. Moreover, Syndegaard et al^23^ presented two specific salivary biomarkers, Prostaglandin (PG) E2 and macrophage inflammatory protein (MIP)-1α, to discriminate gingivitis from healthy periodontium. In their study, levels of interleukin (IL)-1β, IL-6, MMP-8, and PGE2 from 40 periodontally healthy subjects and 40 subjects with gingivitis were measured. While in their study IL-1β failed to discriminate gingivitis from the healthy condition, in the present study, this cytokine was the only statistically significant discriminator identified after a follow-up period of eight weeks.

Specific threshold levels from selected oral fluid biomarkers associated with either gingivitis or periodontitis have not yet been identified.^2^ Both hypo- and hypersalivation could create both false positive or negative interpretations when different concentration levels of biomarkers are being compared. One common approach to overcome this challenge is to analyse and interpret a group of oral fluid biomarkers in association with periodontal health or disease.^2^ So far, only a small number of clinical trials have been able to differentiate between disease and health.^4^ Klukowska et al^8^ assessed the clinical, microbiological, and metabonomic changes in subjects with low and high number of gingival bleeding sites who were submitted to hygiene therapy followed by an experimental gingivitis period. The results revealed a different clinical response between the two assessed groups and no difference in the microbiological analysis profile. The salivary metabonomic analysis in their study demonstrated statistically significant changes in the metabolite composition during study phases associated with plaque toxicity, especially related to the short-chain carboxylic acids propionate and n-butyrate, which tracked clinical changes in gingivitis severity.^8^

## Conclusion

An oscillating-rotating electric toothbrush with an irrigator provided statistically significant reductions in plaque and gingivitis scores over a period of 8 weeks when compared to the use of a regular manual toothbrush alone. These results agree with the available evidence in the literature and are clinically relevant when recommending the use of a powered toothbrush in clinical practice. Moreover, in addition to the clinical assessment of plaque and gingivitis, oral fluid biomarkers derived from WS or GCF for evaluating levels of inflammatory cytokines in larger sample sizes may be able to distinguish small differences between levels of gingivitis and thus be suitable for use in future clinical trials assessing the effectiveness of self-administered oral hygiene products.
